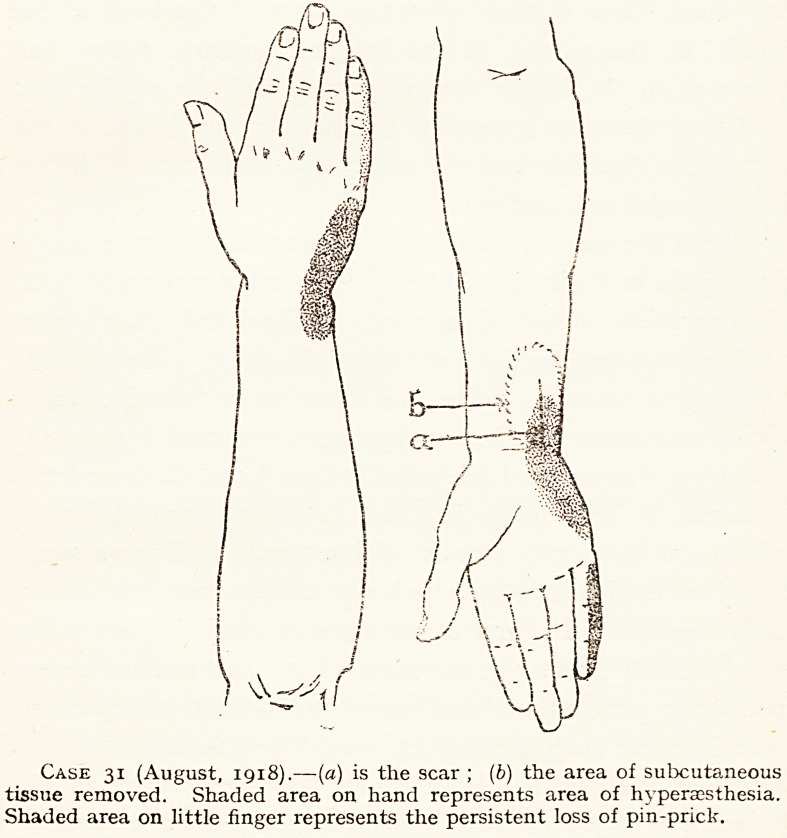# The Operative Findings in Thirty Cases of Gunshot Injury of Nerves, with Remarks on Diagnosis, Localisation, and the Technique of Operation

**Published:** 1919

**Authors:** Charles A. Morton

**Affiliations:** Professor of Surgery in the University of Bristol; Senior Surgeon to the General Hospital, the Children's Hospital, and late Surgeon to the Beaufort War Hospital; and District Consulting Surgeon for Gloucester and Somerset in the Southern Command


### THE OPERATIVE FINDINGS IN THIRTY CASES OF GUNSHOT INJURY OF NERVES, WITH REMARKS ON DIAGNOSIS, LOCALISATION, AND THE TECHNIQUE OF OPERATION.

BY

CHARLES A. MORTON, F.R.C.S.,

*Professor of Surgery in the University of Bristol; Senior Surgeon to the General Hospital, the Children's Hospital, and late Surgeon to the Beaufort War Hospital; and District Consulting Surgeon for Gloucester and Somerset in the Southern Command.*

---

IN cases of complete division of the nerve the ends may lie quite apart, and the proximal end, as is well known, often has quite a marked bulb on it. This bulb is generally adherent by scar tissue to the surrounding structures, as is also the distal end of the nerve. But in some cases of complete division the ends are united by a bridge of scar tissue. This bridge may not at all resemble the nerve in shape, it may (as in one of my cases of divided sciatic nerve) be quite a flat band, but in some cases it may not be at all easy to be sure whether the nerve is actually divided and bridged by scar tissue, or whether what looks like scar tissue is really a continuation of the nerve with scar tissue around, and the nerve thickened by sclerosis. We must not expect to find the divided ends of the nerve always separated by a gap, though we often do, and it is the nature of the intervening tissue, between what is obviously nerve, that is often a difficult matter to determine. A very marked bulb on the proximal side of what seems to be only a bridge of scar tissue would be evidence that the nerve terminated in it, but a bulbous enlargement is often present on an injured nerve which is not completely divided, but would not then have a strand of what appeared to be scar tissue replacing the nerve below it.

It has been recommended that in order to decide if this apparent bridge of scar tissue is really a continuation of the nerve that the surgeon should try and dissect fasciculi through it, but in very dense scar tissue it may not be possible to find them. [1] It has also been stated [2] that the application of a very weak faradic current to the nerve above is a good guide as to whether there are conducting fibres through the scar tissue.

Another very important question to consider is, how to deal with marked thickenings of the nerve. Some surgeons have advised resection of the thickened and enlarged portion of the nerve with union of the ends, [3] others only if the enlarged part of the nerve is hard, [4] and others again [5] only if the conductivity of the nerve to the faradic current is lost or greatly weakened. We must remember that even if the nerve is very greatly thickened recovery may occur. Eve [6] records a case in which the musculo-spinal nerve was as thick as "the end of the little finger," but after freeing the nerve from the surrounding scar tissue recovery quickly took place. My own feeling is that it would be better not to resect unless the nerve were very hard and had lost, or almost lost, faradic conductivity, but that it should be given a further prolonged period for recovery, and then if it did not take place, after the patient had been warned of the long time which must elapse

[1] Eve, *Lancet*, 1915, ii. 1021.

[2] Trotter, *Lancet*, 1915, ii. 1025, and Rowley Bristowe, *Brit. Med. J.*, 1918, i. 6.

[3] Sherren, *Injuries of Nerves*, 1908, p. 78.

[4] McMurray, *Brit. Med. J.*, 1918, i. 380.

[5] Stiles, *Brit. Med. J.*, 1918, i. 380.

[6] *Lancet*, 1915, ii. 1021.

after suture before recovery took place, I think the enlarged portion of the nerve should be excised.

In cases of complete division associated with fracture we may find one or both ends of the nerve lost in the united fracture, and have to chisel them out ; or if the nerve is not completely divided, it may be only bound to the fracture by dense scar tissue, and thus compressed, but it may have to be chiselled out of the callus in which we may find it embedded.

In incomplete lesions (cases in which the nerve is either not completely divided or not divided at all) the most frequent condition found on operation is binding down of the nerve in scar tissue, and in some cases it is flattened from

CASE II. — Epicritic sense impaired and protopathic lost in shaded area.

CASE II.—The thickened part of the nerve, containing the two fragments of metal.

pressure. But either combined with the binding down by scar tissue, or without, there may be fusiform, or less often nodular enlargement of the nerve, and this enlargement is generally firm, but may be soft, and sometimes we find that this enlargement, which usually affects one end of the nerve, contains minute fragments of metal, or stains of metal, showing that they have passed through the nerve. Sometimes the nerve feels as if one or more of its fasciculi had been replaced by a wire. Partial laceration is now and again found—a certain number of fasciculi only are divided, and in one of my cases the lacerating fragment of shell remained embedded in the nerve (Case 15).

CASE I.—To illustrate the presence of a minute fragment of metal in the median nerve.

Some of my cases illustrate what is well known, and is of great practical importance, that in incomplete lesions all the signs of complete division may be present. The lesions usually found in these cases are either pressure of scar tissue or a localised thickening of the nerve itself. However it is unwise to make a diagnosis that the division is complete entirely by the signs which are present, we should wait and see if improvement takes place. From the statements in some books we might conclude that if the "reaction of degeneration" were present, that the lesion must be complete. But I have several times had reports from skilled medical electricians that paralysed muscles had this reaction, and yet those muscles have recovered. On the other hand, if there is faradic irritability, the prognosis as to recovery of the muscle certainly seems good.

But we may also make the opposite mistake and conclude that a complete lesion is incomplete, and go on with electrical treatment and massage for an unnecessarily long time, because we consider we have evidence of a certain amount of function of the nerve. In one of my cases there was an absence of anæsthesia on the dorsum of the foot (Case 19),

CASE 15. — Area of dark shading=loss of both epicritic and protopathic sense. Area of light shading=loss of protopathic sense only.

CASE 15.—Incomplete division of external popliteal nerve by fragment of shell embedded in it.

1. Fragment of metal embedded in nerve.

2. Nerve after removal of fragment of metal, showing laceration of the greater part of the nerve. (*a*) The uninjured strand.

CASE 9. — Gap in median nerve where the ulnar nerve was adherent to it.

which made me think a lesion of the external popliteal was incomplete when the nerve had been completely divided. The mistake was inevitable in this case, for there certainly should have been loss of sensation on the dorsum of the foot in complete division of the nerve. But in injury of this nerve we may be led into error by statements in the ordinary text-books of anatomy. It is there stated that the musculo-cutaneous nerve and the anterior tibial nerve together supply the dorsal aspect of all the toes ; but really, as pointed out by Sherren, [1] the dorsal aspect of all the toes but the great toe is supplied by the plantar nerves, and therefore we must not expect anæsthesia there in even complete lesions of the external popliteal nerves.

In one of my cases of complete division of the ulnar nerve (Case 27) in the middle of the forearm, there was retention of epicritic (but not protopathic) sense on the ulnar side of the ring finger, and on the ulnar side of the palm. The latter may have been due to a very high origin of the palmar cutaneous branch, but the retention of epicritic sense on the ulnar side of the ring finger suggested an incomplete lesion.

In my case of excision of some inches of the posterior tibial nerve (Case 2) there should have been loss of epicritic and protopathic sense on the plantar aspect of the toes (and the dorsal as well), but though they were absent on the sole, yet on the plantar aspect of the great toe he could not only feel a very light touch, but locate it correctly, and was able to feel a prick as distinct from a touch ; and on the plantar aspect of the other toes he could feel a light touch, but could not locate it, and could not distinguish a prick from a touch. This retention of sensation might have been very misleading in the diagnosis between a complete and an incomplete lesion.

It has been stated by Sherren [2] that division of the musculo-spiral nerve below the origin of its external cutaneous branches does not cause anæsthesia in the so-called "radial supply," as would be expected from statements in the text-books, but that the "radial supply" depends on the long external cutaneous branch of the musculo-spiral, and

[1] *Injuries of Nerves*, 1918. [2] *Injuries of Nerves*, p. 237.

the posterior branch of the external cutaneous which join the radial. I found this to be so in one of my cases. The musculo-spiral nerve was involved in a fracture at the middle of the humerus (Case 24). But in some other cases there have been areas of both epicritic and protopathic loss in some part of the "radial supply" (see chart of Case 12), and in these cases the external cutaneous branches of the musculo-spiral and the posterior branch of the external cutaneous were not injured. In one case a fragment of metal was actually lodged within the nerve, three inches above the external condyle and an area of anæsthesia was produced as shown in the chart (see chart of Case 11), and pressure on a foreign body in the nerve at this level caused pain in the "radial supply" of the thumb and index finger.

CASE 12.—Loss of epicritic sense in light shading and pin-prick in dark.

Sherren also says that the upper part of the radial nerve may be excised for grafting without producing any sensory loss. I removed two and a half inches to graft into another nerve. The sensory loss is shown in chart of Case 27, and is almost exactly the radial supply of the anatomy text-books (the loss of sensation in the ulnar supply was due, of course, to the original lesion). In Case 29 there had been a fracture of the upper end of the radius, and the radial nerve was probably injured also. There was a little epicritic and protopathicloss on the dorsal aspect of the ·humb (as shown in chart), but there was also pain in thesame nerve distribution, and this was clearly due to an injury of a branch of the external cutaneous nerve, for when pressure was made on a scar over it it caused the pain, and resection of the branch with the scar tissue in which it was involved cured it. But

CASE 27.—The light shading is the area of loss of both epicritic and protopathic sense, and the smal area of dark shading the loss of protopathic sense beyond the area of loss of epicritic sense.

CASE 29.

that the lesion of the branch was not the cause of the anæsthesia on the back of the thumb was shown by the fact that excision of the branch produced another area of anæsthesia near the wrist and the centre of the hand. There was a scar on the posterior aspect of the forearm, which might have involved the long external cutaneous branch of the musculo-spiral nerve, but the great probability is that the radial nerve was damaged at the seat of fracture of the radius. The case is not, of course, so conclusive as the one of excision of the upper end of the radial nerve, but is fairly good evidence.

Not only may we be misled as to the diagnosis between a complete and an incomplete lesion by the absence of anæsthesia where we might expect it, we may also be misled by the retention of power to perform some movement which we consider should be lost in complete division of the nerve. I have twice been misled by this, in complete division of the ulnar nerve, with persistent ability to adduct the thumb—not the false adduction which Sherren so clearly describes[1] due to action of either the long flexor or the extensors, but the true adduction he describes, and such movement as you can produce by electrical stimulation of the adductor. In one case I was quite misled by the return of this action of adduction, and demonstrated to a surgery class that it was true and not false adduction, and that the lesion of the ulnar nerve must therefore be incomplete. Yet the ulnar nerve was wholly divided (Case 27). I have found the same condition in another case of divided ulnar nerve (Case 20) since my experience in Case 27, but taught by experience in that case I was not led into an error in diagnosis. I believe the adduction is carried out by the extensor longus pollicis (extensor secundi internodii pollicis). Another mistake which may be made in the diagnosis between a complete and an incomplete lesion of the ulnar nerve is that the flexor carpi ulnaris is producing ulnar flexure of the wrist, when it is paralysed. I have seen this action in cases of complete division of the ulnar nerve. Mr. Sherren has called attention to this,[2] and he considers the action is performed by the palmaris longus. Where I have found ulnar flexion in

[1] *Injuries of Nerves*, p. 248. [2] *Injuries of Nerves*, p. 247.

complete division of the ulnar nerve it has seemed to me to be due to a combined action of the flexor carpi radialis and the extensor carpi ulnaris. A knowledge of the substitution of the action of a muscle or group of muscles for a paralysed muscle or group of muscles is of great importance in the diagnosis of nerve lesions, and it may be well to call attention to two more instances of it. Sherren[1] has pointed out that when the normal flexions of the elbow are paralysed flexion may be performed by the supinator longus (brachio-radialis) and the extensors of the wrist, when the forearm is fully pronated. And Souttar and Twining[2] have pointed out that in complete lesions of the median and ulnar nerve flexion of the wrist can still be performed by the "short extensors of the thumb."

But when we have made the diagnosis of an incomplete lesion, we must not therefore conclude that recovery will be only a matter of time. In some of these incomplete lesions some of the fasciculi of the nerve are completely divided, and only operation for their reunion will lead to recovery. Therefore if there is no return of power or sensation in some of the affected areas of supply after a prolonged period of suitable electrical treatment and massage, operation must be undertaken. Case 12 is a good illustration of the need for this.

In two of my cases (Cases 1 and 9) the lesions suggest the locality of fibres within the median nerve supplying certain muscles and areas of skin. In Case 9, in which there was division of the inner part of the median nerve in the axilla, there was a loss of epicritic and protopathic sense in the cleft between the ring finger and middle finger, and on the radial side of the middle finger only, with paralysis of the abductor pollicis only. In Case 1 there was a foreign body in the

[1] *Injuries of Nerves*, pp. 197, 231.

[2] *Brit. J. of Surg.*, October, 1918, p. 282.

outer half of the median nerve just above the elbow, and there was paralysis of the flexor longus pollicis only, and the epicritic sense was present, but feeble and tingling in the median supply in the fingers and thumb, and protopathic sense was not lost there. These two cases taken together seem to suggest that the fibres for the flexor longus pollicis pass down the outer and those for the intrinsic muscles of the hand the inner side of the nerve, and that sensation is more affected by a lesion of the inner than the outer; but we must remember that the lesions were at different levels, the one with the greater sensory change was in the axilla, and the one with the very partial sensory change at the level of the elbow.

In connection with the diagnosis of the nature of the lesion of an injured nerve, we have to recognise the disconcerting fact that with extensive paralysis and anæsthesia the nerve may on exploration be found to naked-eye inspection and on palpation normal. This was so in a case in which the musculo-spiral nerve was examined, and other surgeons have had the same experience with this nerve.[1] In another of my cases of gross lesion of the musculo-spiral nerve (Case 12) there were signs of a partial lesion of the median and ulnar nerves, which had persisted for several months and up to the time of operation, and yet these nerves appeared perfectly normal when exposed. The signs present were patchy epicritic and protopathic loss on the median and ulnar supply of the fingers, and very feeble action of the flexors of the wrist and fingers, and of the intrinsic muscles of the hand. I had the same experience in a case of divided external popliteal nerve (see chart Case 21). In this case not only were the calf muscles very feeble, but there was epicritic and protopathic loss in the distribution of the internal popliteal nerve, suggesting a partialr lesion of it as well as the

[1] Michell Clarke and Short, *Bristol M.-Chir. J.*, July, 1917.

complete lesion of the external popliteal, but when the external nerve was sutured four months after the injury the internal one was found to be uninjured. The lesion of the internal popliteal nerve must have been a condition of nerve

CASE 21.—Epicritic and protopathic loss in shaded area. Dotted area = uncertain epicritic loss and no protopathic loss.

concussion only. We must not, therefore, conclude that in all cases a marked feebleness of the opposing group of muscles to the group paralysed, even when combined with anæsthesia, indicates a complex gross lesion of the nerves. I have included in this series of cases one (Case 3) which was not a nerve operation. It was an operation for secondary hemorrhage in the popliteal space, but I was able to find out the condition of the internal popliteal nerve. There was both epicritic and protopathic loss on the plantar aspect of all the toes, and the dorsal aspect of the outer three, and patchy loss on the sole. But the nerve was not even exposed in the wound. The sensation quickly returned.

In cases in which there is more than one scar in the course of a nerve the function of which is impaired it may be difficult to decide at which level the nerve has been injured. The involvement or escape of branches to certain muscles may enable us to decide, but will not always do so. We may be misled in such cases by pressure on the scar causing tingling in the distribution of the nerve. In one of my cases (Case 5) there were two scars at the back of the thigh in the course of the internal popliteal nerve, and pressure on the higher caused pain in the sole of the foot, and was therefore supposed to be over the lesion in the nerve, but the lesion of the nerve was found several inches lower, under another scar.

I had five cases in which gunshot wounds of nerves caused great pain in their distribution. Two cases recovered without operation—lesion of the posterior tibial and ulnar nerves, and three cases required operation (Cases 2, 4 and 5). In these three cases the nerve was found to be bound down in dense scar tissue in Case 2, apparently injured by a small fragment of metal, and thickened in Case 5, and thickened and adherent to scar tissue in Case 4. These were all lesions which are frequently present without pain, and there seems to be no evidence to show why in certain cases the severe pain is caused. In three of my cases in which foreign bodies were actually present in the nerves there was not any severe pain—the patients made no complaint of pain—and yet one would expect such a condition would be the most frequent cause of such pain. How much less likely then is the pressure of a foreign body lying merely against the nerve to cause some pain. There was no skiagraphic evidence of any foreign body near the nerve in the two cases I did not operate on.

Limitation of space prevents my writing of the treatment of these distressing cases at any length now, but I should like to say that I have found injection of alcohol in the nerve completely relieve the pain in two cases in which I practised it, and the functional loss which results is said[1] to be only temporary, but may last many months.

I have had one case of very intractable hyperæsthesia in a small area of the supply of the ulnar nerve, which was injured just, above the wrist (see chart Case 31). The chart

CASE 31 (August, 1918).—(*a*) is the scar; (*b*) the area of subcutaneous tissue removed. Shaded area on hand represents area of hyperæsthesia. Shaded area on little finger represents the persistent loss of pin-prick.

[1] Sicard, *Lancet*, 1918, i. 213.

shows the area of hyperæsthesia, which was so great that the patient could not bear the slightest touch on it. I explored the nerve at the seat of injury, and found the common lesion of thickening and binding down in scar tissue. The freeing of the nerve led to recovery of power in the paralysed intrinsic muscles of the hand, but had no effect on the hyperæsthesia. This was in the distribution of the palmar cutaneous branch, and the dorsal branch, but there was no evidence of injury to the nerve above the level at which they arise. I operated again, and as I could not on a second attempt identify the palmar cutaneous branch, I dissected out an area of subcutaneous tissue which must have included it, but again I failed to relieve the hyperæsthesia.

I do not propose to write at any length on the technique of the operation for dealing with injured nerves, but there are a few points I should like to allude to. One is the statement that the ulnar nerve can be elongated by bringing it to the front of the internal condyle. I was not myself able to see how elongation could be brought about in this way, and I therefore experimented on the dead body. I found that it did not make any difference to the length of the nerve, whether it ran behind or in front of the condyle, in either the flexed or extended positions of the forearm, but that the mere freeing of the nerve from its connective tissue bed behind the condyle allowed of considerable elongation.

I have already published[1] a case of suture of a divided fasciculus of the musculo-spiral nerve, and I would again call attention to the desirability of treating all partial divisions of a nerve in this way.

With regard to the debated questions as to whether the sutures should pass through the nerve, or only through its sheath, whether it is better to wrap the nerve in Cargile membrane or fat or fascia, and as to the value of nerve grafting

[1] *Lancet*, 1918, i. 373

or attachment to other nerves, only observation of cases over a very prolonged period of time can decide, and it is so very difficult to do this in the case of military patients whose homes are elsewhere. With regard to the practice of wrapping the nerve in Cargile membrane, I can say that I have never had any trouble from it, as I have had in operation on tendons, from a late discharge of the membrane, and I have used it in all my nerve cases. In one case (Case 4) in which I had to operate again a month after the first operation I found it still wrapped around the nerve, though very fragile. It was chromicised.

It is well to recognise that a gap of three inches in the sciatic nerve (Case 23) can be quite well closed without tension, by nerve stretching, and flexion of the leg on the thigh.

I suppose many of us are rather sceptical as to very rapid return of sensation after operation on a nerve, but I have had one undoubted case (Case 6) of return of epicritic sense in the median supply within twenty-four hours after freeing the nerve from the dense scar tissue in which it was embedded.

### INCOMPLETE INJURIES (NOT FRACTURES).

| NAME, ETC. HOSPITAL. | NERVE. | SIGNS AND SYMPTOMS. | FINDING AT OPERATION. |
|---|---|---|---|
| 1<br>J. M. G.,<br>æt. 35.<br>Nov., 1917.<br>B.W.H.* | Median. | Wounded two months before admission, and had small scar of punctured wound in course of median nerve two inches above elbow, and skiagram showed a minute foreign body there. Epicritic sense in median supply on fingers and thumb not absent, but dull and tingling. No protopathic loss, only loss of power in flexor long pollicis. Pressure on foreign body caused tingling in centre of flexor aspect of forearm, and in index and middle fingers. | A minute fragment of shell was found in the outer half of the nerve, which was enlarged so as to be spindle-shaped at seat of injury. It lay quite within the nerve, and had to be removed by a longitudinal incision in the nerve. See diagram. |
| 2<br>T. H. C.,<br>æt. 19.<br>B.W.H.<br>Nov., 1917. | Posterior tibial. | Very severe pain in distribution of nerve. Large granulating wound, involving the deep structures just below middle of calf of six weeks' duration. Very severe pain in sole of foot. No epicritic loss, and pin-prick causes considerable pain. | Posterior tibial nerve was found flattened and bound down by dense scar tissue to back of tibia. It was freed and wrapped in cargile membrane. The immediate result of this was that the pain was very much relieved, and was almost confined to the distribution of the external plantar, and the hyperæsthesia disappeared. The relief continued for a fortnight; then as the pain began to get so severe again that frequent hypodermic injections of morphia were required, on December 15th I operated again, and found the nerve again involved in dense scar tissue. I excised $5\frac{1}{4}$ in. The nerve where it had been bound down by the scar tissue was not enlarged, but on one aspect (probably the one corresponding to the course of the fibres of the external plantar nerve) was very indurated and contained fewer nerve bundles than above and below. The operation quite cured the pain. |

| Name, etc. Hospital. | Nerve. | Signs and Symptoms. | Finding at Operation. |
|---|---|---|---|
| **3** McC. B.W.H., Sept., 1918. | Internal popliteal nerve. | I have included this case though the operation was not for the nerve lesion as it is very instructive, as showing how little damage may be done to a nerve, and yet there may be anæsthesia. There was both epicritic and protopathic loss on plantar aspect of all the toes, and dorsal aspect of the outer three, and patchy loss on sole. | The popliteal space had to be opened up for secondary hemorrhage, but the internal popliteal nerve was not exposed in the wound, or if exposed at all only a very minute area of it. But I think it was not exposed. Of course it is just possible that a very minute piece of metal may have penetrated the nerve, but the anæsthesia completely disappeared in a month, so that this could hardly have occurred. |
| **4** W. H., æt. 24. B.W.H., April, 1918. | Internal popliteal | Severe pain in distribution of nerve. Severe pain in sole, heel, and plantar aspect of toes. Came on a few minutes after wound in region of internal popliteal nerve. There was loss of epicritic and protopathic sense in front part of sole and protopathic on plantar aspect of all toes but great toe. Skiagram showed several minute fragments of metal in region of nerve. The sole was hyperæsthetic. | There was a fusiform enlargement on the nerve, and a few black spots on it as if minute fragments of metal might be embedded in it, but a longitudinal incision in the nerve did not discover any. There was some scar tissue on it, but it was not compressed by scar tissue. The pain was only relieved for a few days. The area of anæsthesia remained the same, but the area of hyperæsthesia shifted to the outer part of the foot. The pain was chiefly in the heel. It became so severe again he had to have frequent hypodermic injections of morphia. I then injected the nerve with alcohol with success. The same thickening of the nerve was then found as at the first operation, one month later. |
| **5** S., B.W.H., April, 1918. | Internal popliteal. | Severe pain in distribution of nerve. Track of gunshot wound through popliteal space. Immediately he received wound he felt tingling in foot, and very soon after pain there. A month later he was admitted suffering from severe pain starting in heel and passing forward over sole, which required hypodermic injection of morphia. There was loss of epicritic and protopathic sense on heel, but no hyperæsthesia. | Operation five weeks after injury. Nerve was found much thickened for one inch and very adherent to dense scar tissue on its anterior aspect, where a minute boss protruded from it. It was injected with alcohol with complete relief of the pain. |

| | | | |
|---|---|---|---|
| 6<br>F. C. B.,<br>æt. 25.<br>G.H.†<br>June, 1918. | Median. | Wounded ten months before. Scar in portion of median nerve at middle of forearm. Epicritic sense either lost or impaired in median supply on fingers and thumb, and protopathic loss in same area except thumb and radial side of ring finger. There was jerky feeble flexion of last joint of thumb and index finger, but flexion was fairly good in all other joints of the fingers. Abductor pollicis was paralysed. There seemed to be a partial lesion of the median, probably below anterior interosseous branch, which might be partly injured also, but the feeble action of some of the flexors might be accounted for by direct muscle injury. | One inch of median nerve was involved by such a dense scar adherent to interosseous membrane and the bones, that it felt like a lump of bone. The nerve was dissected out of this, and was not itself thickened. The feeble flexors were also involved in scar, and the lesion was below the region of the anterior interosseous nerve. |

* Beaufort War Hospital.
† General Hospital.

### INCOMPLETE LESIONS.

| | | | |
|---|---|---|---|
| 7<br>F. K.,<br>æt. 32.<br>B.W.H.,<br>Oct., 1916. | Musculo-spiral. | Signs of complete division of nerve. Scar in upper third of arm. Area of anæsthesia on back of forearm, and part of "radial supply" on hand, on dorsal aspect of thumb, but only over dorsal aspect of metatarsal bone of index finger. | Operation three months after injury. Nerve incorporated in aneurysmal sac of axillary artery and flattened. |
| 8<br>A. C.,<br>æt. 21.<br>B.W.H.,<br>Dec., 1917. | Ulnar nerve. | Complete paralysis of ulnar nerve from wound in upper part of arm. No faradic and very feeble galvanic reaction. In many of the muscles there was polar reversal. No improvement in nine months. | Operation nine months after injury. One inch of nerve was involved in dense scar tissue. When freed it seemed to have been divided and united by scar tissue. This was excised and the ends united, but on examination after resection I could trace a few nerve fibres running through it, though it was almost wholly fibrous tissue stained by metal. |

| Name, etc. Hospital. | Nerve. | Signs and Symptoms. | Finding at Operation. |
|---|---|---|---|
| 9<br>G. D.,<br>æt. 21.<br>B.W.H.,<br>Mar., 1917. | Median. | Anæsthesia of cleft between middle and ring finger and on other side of end of middle finger with paralysis of abductor pollicis. Pressure on the nerve in the arm caused tingling in median supply on finger. | At operation I found destruction of the inner part of the median nerve and adhesion of end of ulnar nerve to it. See diagram. |
| 10<br>H. G. B.,<br>æt. 30.<br>B.W.H.,<br>April, 1917. | Recurrent laryngeal nerve. | Struck in neck by fragment of shell on April 6th. Immediate loss of voice. Much hemorrhage from wound. When admitted to B.W. Hospital at end of April voice was hardly more than a whisper. Laryngeal examination showed only very imperfect action of left cord. There was a small, dry wound over anterior border of left sterno-mastoid about middle of the muscle, with induration of the underlying tissue. A skiagram showed a fragment of shell about $\frac{1}{3}$ inch in diameter lying just to the left of middle line, and about one inch above the top of the sternum. | It was impossible to say to what extent the recurrent nerve was injured, but the voice was improving by May 23rd, and by then the wound had quite healed. I thought the presence of the foreign body might tend to retard or prevent recovery, and on May 23rd I removed it. It lay on the left edge of the œsophagus, fixed to the spine, and close to the recurrent laryngeal nerve, but did not actually press on it. The recurrent nerve was fully exposed up to the point where it runs under the inferior constrictor of the pharynx, and was quite normal. Although the nerve was not itself pressed on by the scar tissue, all the parts around were much bound down by it, and their release may have had some effect on the nerve. After the operation there was an immediate return of tone in his voice. His larynx was examined by Major Watson Williams on June 23rd, when his voice had greatly improved. He thought there had been complete paralysis of the left cord, and that the adductor was recovering The patient was then suffering from some breathlessness on exertion, which Major Watson Williams considered to be due to abductor paralysis. |

| | | | |
|---|---|---|---|
| **11** T. W., æt. 20. B.W.H., July, 1917. | Musculo-spiral. | Complete paralysis of long extensors of fingers and extensors of thumb, but feeble action of extension of wrist, probably because lesion is below branch to extensor carpi radialis long. The action of the wrist extensors became quite strong, but there was no return of power in extensors of fingers or thumb. Sensation lost in area of "radial supply" (see chart). This did not return. The nerve was injured at outer border of triceps, three inches above external condyle, and was here thickened, and pressure on it caused tingling in "radial supply." Two minute fragments of metal were seen in a skiagram to lie near the nerve, but it was not evident that they lay within it. The paralysed muscles did not react to faradism, but they did feebly to galvanism. | Operation six months after injury showed a spindle-shaped enlargement of the nerve, one inch long, and very firm, containing the two fragments of metal. They were quite within the sheath of the nerve (see diagram). The enlarged portion of the nerve was firmly bound down by scar tissue. It was possible to remove the fragments of metal (which were quite thin) without damage to the nerve by a minute longitudinal incision. |
| **12** B., æt. 19. B.W.H., Sept., 1917. | Musculo-spiral. | Injury at level of one inch below anterior axillary fold. Complete paralysis of extensors of wrist and fingers and thumb, and incomplete of triceps. But flexor muscles of hand and fingers and intrinsic muscles of hand very feeble. Patchy epicritic and protopathic loss on palmar aspect of hand and fingers, so that median and ulnar nerves must be slightly damaged. Area of anæsthesia on dorsum as shown in chart. The explanation of the loss of epicritic sense over the whole dorsum except the ulnar supply is due to partial lesion of median nerve. The electrical report was that all the muscles supplied by musculo-spiral nerve below the triceps show the R.D. | Operation four months after injury, as there was no improvement in muscles supplied by musculo-spiral nerve. Division of an anterior fasciculus of the nerve was found, and the whole nerve was bound down by dense scar tissue. The condition found at operation and the union of the fasciculus is described in the *Lancet*, 1918, i. 373. The median and ulnar nerves were quite normal, and were not at all bound down by scar tissue. |

### INCOMPLETE INJURIES.

| Name, etc. Hospital. | Nerve. | Signs and Symptoms. | Finding at Operation. |
|---|---|---|---|
| **13** W. H., æt. 22. B.W.H., May, 1917. | Ulnar musculo-spiral and internal cutaneous. | Healed track of foreign body through axilla. At first complete paralysis of all muscles supplied by musculo-spiral and ulnar nerves. Six months later there was recovery of power in triceps, and some return of power in extensors of wrist and thumb, but the paralysis of the ulnar muscles remained complete. There was both epicritic and protopathic loss in the ulnar supply. There was also a strip of epicritic loss on the inside of the forearm, chiefly on its posterior aspect, extending half-way from wrist to elbow. There was at first no faradic irritability in any of the paralysed muscles, and a very slight irritability to the constant current, and almost equal action with either pole. There was no sign of any definite lesion of the median nerve. | Operation six months after injury. The ulnar, musculo-spiral, internal cutaneous and median, were all somewhat involved in scar tissue, but the ulnar was bound down by dense scar tissue, and contained two small nodular enlargements. The musculo-spiral was the one most involved in scar tissue after the ulnar. |
| **14** J. C., æt. 35. B.W.H., May, 1917. | Musculo-spiral. | Scar from gunshot wound in axilla. Complete paralysis of extensors of fingers and thumb and wrist, and partial of triceps. No faradic reaction in extensors of wrist or finger, but feeble in triceps. Only very feeble reaction to constant current in extensors below triceps. No certain epicritic loss, but protopathic over dorsum of thumb and second metacarpal bone. No recovery four months after operation. | Operation four months after injury. Musculo-spiral nerve exposed over whole area in which it could have been wounded, *i.e.* both in axilla and at back of arm, and the only abnormal condition found was that the nerve felt broader than usual just as it passed to the back of the arm, and it also felt as if there were a wire running through it, as if some fasciculus had become densely sclerosed. But there was no scar tissue immediately around it, but a good deal in the surrounding parts. |

| | | | |
|---|---|---|---|
| **15**<br>J. H. C.,<br>æt. 35.<br>B.W.H.,<br>Nov., 1918. | External popliteal. | *Several small fragments of shell penetrated upper* and outer part of leg, and one seen in skiagram to be in position of external popliteal nerve, where it passed by head of tibia. Paralysis of extensor long digit, extensor long hallucis, and peronei, but good power in tibialis anticus, with area of anæsthesia shown in chart. Diagnosis of partial lesion of external popliteal nerve leaving fasciculi to tibialis anticus uninjured. | *Operation two and a half months after injury. Fragment of metal one centimetre by half a centimetre* embedded in external popliteal nerve, having divided two-thirds of its fibres. The divided portion of nerve was sutured. The fibres which had not been divided were the anterior fibres, *i.e.* the deep fibres as the nerve lay exposed in the wound.<br>See Fig. |

COMPLETE LESIONS.

| | | | |
|---|---|---|---|
| **16**<br>G. M.,<br>æt. 20.<br>B.W.H.,<br>Dec., 1915. | Sciatic. | Divided by passage of rifle bullet across middle of thigh three months before operation. The typical paralysis and anæsthesia were present. There was no faradic action and very feeble galvanic action in the paralysed muscles, and an equal response with either pole. | Both ends of the nerve were involved in a dense mass of scar tissue, and separated by a strand of half inch of scar tissue, in which no nerve fibres could be discovered on careful examination after resection. |
| **17**<br>G. D.,<br>æt. 21.<br>Mar., 1917. | Ulnar and internal cutaneous. | Scar of wound in axilla. Complete loss of function in ulnar, with loss of faradic and very feeble galvanic reaction, with also strip of anæsthesia along inner aspect of forearm. There were also signs of an incomplete lesion of the median, and the combination suggested a lesion of the inner cord of the brachial plexus, but were not quite the same. Pressure on the ulnar nerve in the arm caused tingling over the dorsal surface of the metacarpo-phalangeal joints of ring and little fingers. | At operation complete division of the ulnar and internal cutaneous nerves was found. |
| **18**<br>L.<br>B.W.H.,<br>Aug., 1917. | External popliteal nerve. | Complete paralysis of muscles supplied, and usual sensory loss. No faradic reaction and sluggish reaction to galvanism. K.C.C.=A.C.C. | At operation complete division of the nerve was found, with bulb on upper end, and both ends involved in scar tissue. |

| Name, etc. Hospital. | Nerve. | Signs and Symptoms. | Finding at Operation. |
|---|---|---|---|
| **19** H. H., æt. 23. B.W.H., Feb., 1918. | External popliteal nerve. | Paralysis of dorsiflexors of ankle, and extensor of toes and peronei, but on repeated examination no epicritic or protopathic loss could be discovered on dorsum of foot, and I therefore concluded it must be only a partial lesion. The paralysed muscles did not react to faradism, and only feebly to galvanism. | The ends of the external popliteal nerve, jnst above the head of the fibula, were separated by one inch of flat scar tissue containing no nerve fibres. The operation was seven months after the injury. |
| **20** J. W., æt. 30. B.W.H., June, 1918. | Ulnar. | Scar over ulnar nerve just where it passes through the heads of the flexor carpi ulnaris. Typical signs of lesion of ulnar nerve just below elbow, except that the power of adduction of thumb and ulnar flexion of wrist were retained. | Nerve found completely divided. The upper end had a large bulb on it, the lower was merged in the neighbouring muscle. |
| **21** W. D., æt. 36. B.W.H., July, 1918. | External popliteal. | Scar of wound just above head of fibula, on posterior aspect. Complete paralysis of muscles supplied by the external popliteal nerve, and epicritic and protopathic loss in its supply. But both epicritic and protopathic sense were also less in area of internal popliteal supply, though nothing abnormal was discovered in the nerve at the operation. The calf muscles acted feebly. See chart. | Operation four months after injury. Nerve completely divided. The upper end of the nerve terminated in a bulb, the lower was not enlarged, and adherent in scar tissue just behind head of fibula. |
| **22** F. C. B.W.H., Sept., 1918. | Median and ulnar. | Both nerves divided in large open wound just above the wrist, in April, 1918, with loss of power in intrinsic muscle of hand, usual ulnar and median anæsthesia. | Operation six months after injury. The ends of both nerves were involved in scar tissue. Neither nerve was distinctly bulbous. |
| **23** S. H., æt. 25. B.W.H., Mar., 1918. | Sciatic. | Scars of small gunshot wounds at back of thigh, Typical paralysis below branches to hamstrings, with no reaction to faradism but feeble to galvanism, with equal effect with either pole. Usual area of anæsthesia, | At operation the ends were found separated, and with a bulb on the upper. |

INJURIES OF NERVES ASSOCIATED WITH FRACTURE (COMPLETE LESIONS).

| | | | |
|---|---|---|---|
| **24**<br>A. K.,<br>æt. 19.<br>G.H.,<br>July, 1915. | Musculo-spiral. | Fracture of middle of humerus. Complete paralysis of muscles supplied by nerve, but no sensory lesion in so-called "radial area." | Operation three months after injury. Complete division. Proximal end ran into united fracture and had to be chiselled out. Distal end adherent by dense scar tissue to bone at a lower level. |
| **25**<br>T. R.,<br>æt. 35.<br>B.W.H.,<br>Jan., 1916. | Ulnar. | Fracture of lower end of humerus, with complete paralysis and anæsthesia in ulnar supply, There was no faradic reaction, and very feeble galvanic, and the effect of the two poles was equal. | At operation, two and a half months after the injury, the nerve was found densely adherent to the humerus at seat of fracture, and a small fragment of bone had to be removed to free it. The ends were united by a narrow bridge of fibrous tissue only. Both ends of the nerve were considerably thickened, but there was no bulb. |
| **26**<br>P. P.,<br>æt. 39.<br>G.H.,<br>April, 1917. | Musculo-spiral. | Fracture of lower end of humerus, followed by necrosis, for which sequestrotomy had to be performed, so that operation could not be undertaken for two years after injury. There was complete paralysis of all muscles supplied by nerve below seat of lesion. | Nerve was found completely divided. and both ends involved in scar tissue, at the site of the old fracture. There was a marked bulb on the proximal end. |
| **27**<br>E. B.,<br>B.W.H.,<br>May, 1917. | Ulnar. | Fracture middle of ulnar. The signs suggested an incomplete lesion as the anæsthesia was not complete, and the adductor of thumb was active. | Operation one year after injury. Complete division was found, with a marked bulb on proximal end. The ends were bound down in scar tissue. |
| **28**<br>A. V. C.,<br>æt. 30.<br>B.W.H.,<br>Nov., 1918. | Median. | Wounded May 4th, 1918, just above wrist. Typical anæsthesia of division of median nerve here, and complete loss of power, with marked wasting in abductor pollicis. | November, 1918. Median found completely divided and both ends bulbous, but the distal end rather more than the proximal. The ends were united by a thin strand of tissue about one inch in length. The nerve had been divided just before passing under the annular ligament, but only one tendon was divided. |

### INJURIES OF NERVES ASSOCIATED WITH FRACTURES (INCOMPLETE LESIONS).

| NAME, ETC. HOSPITAL. | NERVE. | SIGNS AND SYMPTOMS. | FINDING AT OPERATION. |
|---|---|---|---|
| **29** M. H., æt. 44. B.W.H., Nov., 1915. | Radial and external cutaneous. | United fracture of radius, near lower end. Epicritic and protopathic loss on dorsum of thumb, and pain there. Pressure on scar in course of external cutaneous nerve in forearm also caused pain in same area. | The pain was cured by excision of branch of external cutaneous nerve which was involved in the scar, together with the scar. |
| **30** W. M., æt. 27. B.W.H., Oct., 1916. | Musculo-spiral. | Fracture of upper end of humerus. Complete paralysis of extensors of wrist, thunb, and long extensors of fingers. No return of power in a year. | Nerve then exposed at seat of injury and no definite lesion found. Power was gradually returning in paralysed muscles during the following year. |
| **31** B., æt. 24. B.W.H., Feb., 1918. | Ulnar. | Fracture of lower end of ulnar in July, 1917. Scar just above wrist. In October, 1917, it was noted that there was epicritic and pin-prick loss in ulnar supply on hand and in little finger, and in November an area of marked hyperæsthesia in ulnar supply on hand and wrist was found. In February, 1918, the hyperæsthesia persisted, but there was no longer epicritic loss in little finger, but pin-prick loss continued there. The intrinsic muscles of the little finger were paralysed, but he could adduct thumb. Electrical treatment failed to relieve the hyperæsthesia. | Operation nine months after injury. The ulnar nerve was enlarged for half an inch under the scar just above the wrist. It was much thickened and bound down by scar tissue. It was freed and wrapped in cargil membrane. I could not identify the cutaneous palmar branch which came off above the lesion. The operation failed to relieve the hyperæsthesia, but when examined four months later the intrinsic muscles of the little finger had regained some power, and the loss of pin-prick was only on the ulnar side of the little finger. On August 7th, 1918, I again searched for the palmar cutaneous branch, but failed to find it. I resected the scar and the surrounding subcutaneous tissue for some inches, so that this branch must have been removed. I found the ulnar nerve still swollen. This operation did not relieve the hyperæsthesia. |

## Figures and Tables

**Case II. f1:**
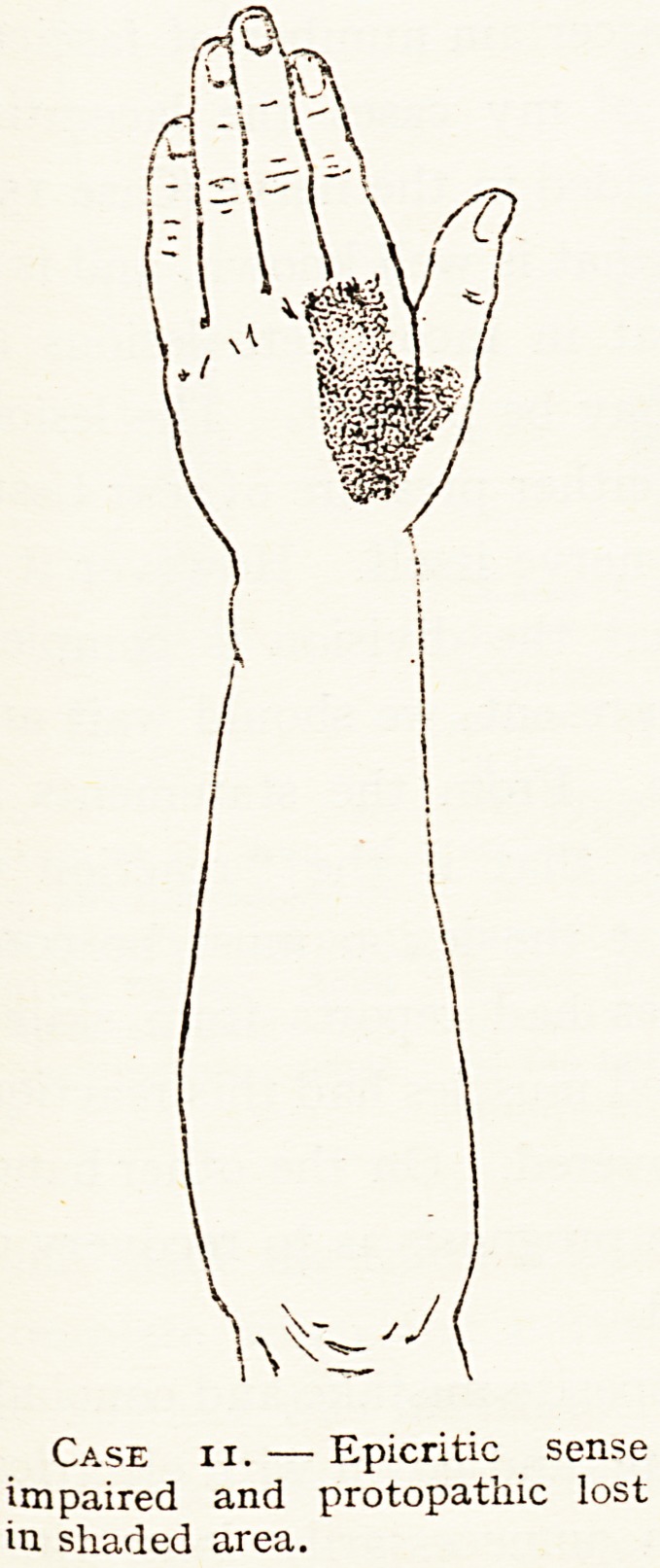


**Case II. f2:**
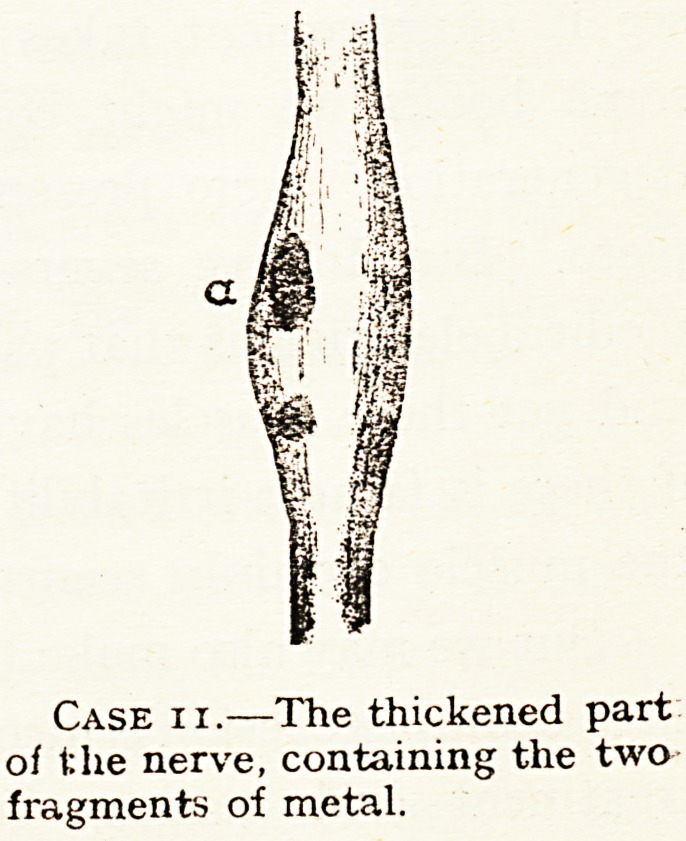


**Case I. f3:**
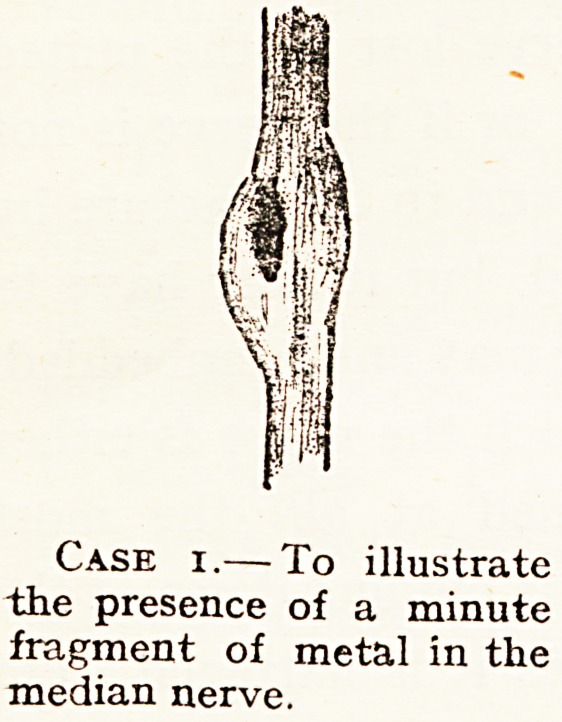


**Case 15. f4:**
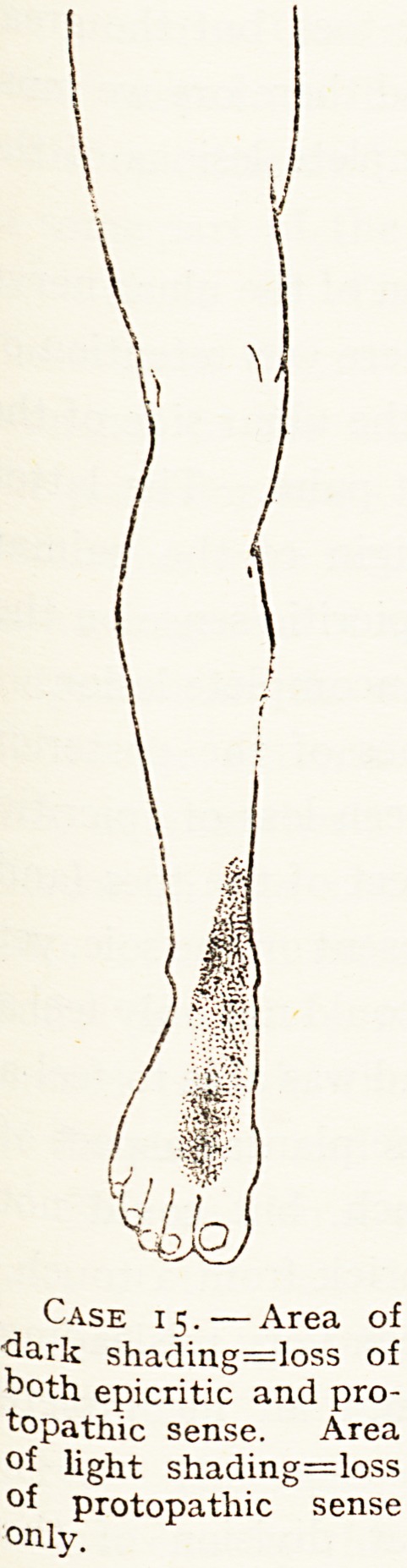


**Case 15. f5:**
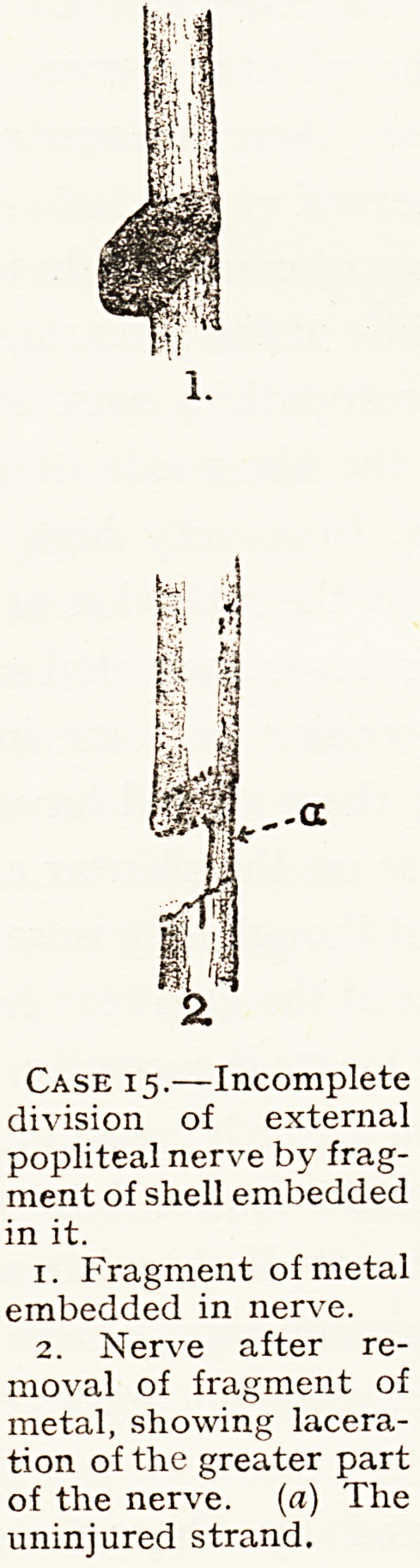


**Case 9. f6:**
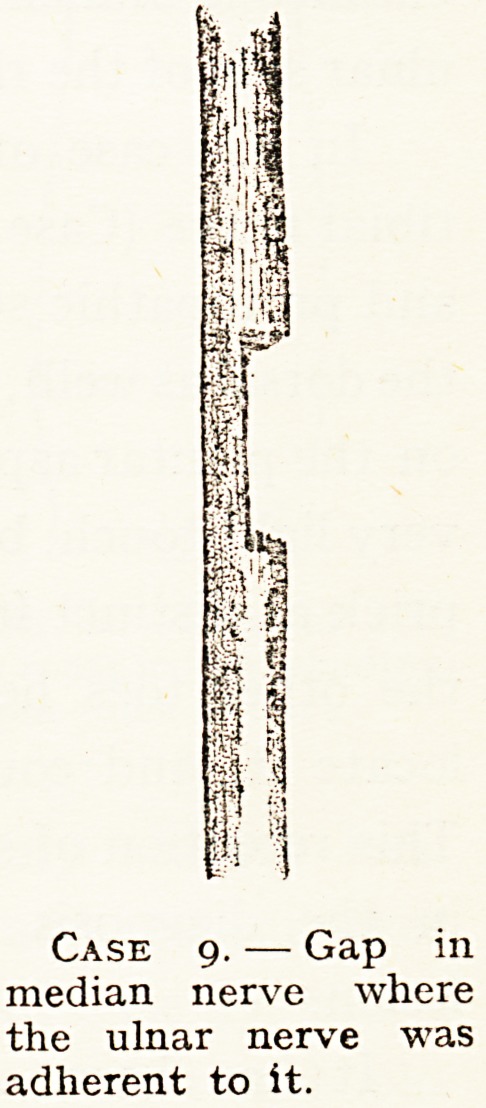


**Case 12. f7:**
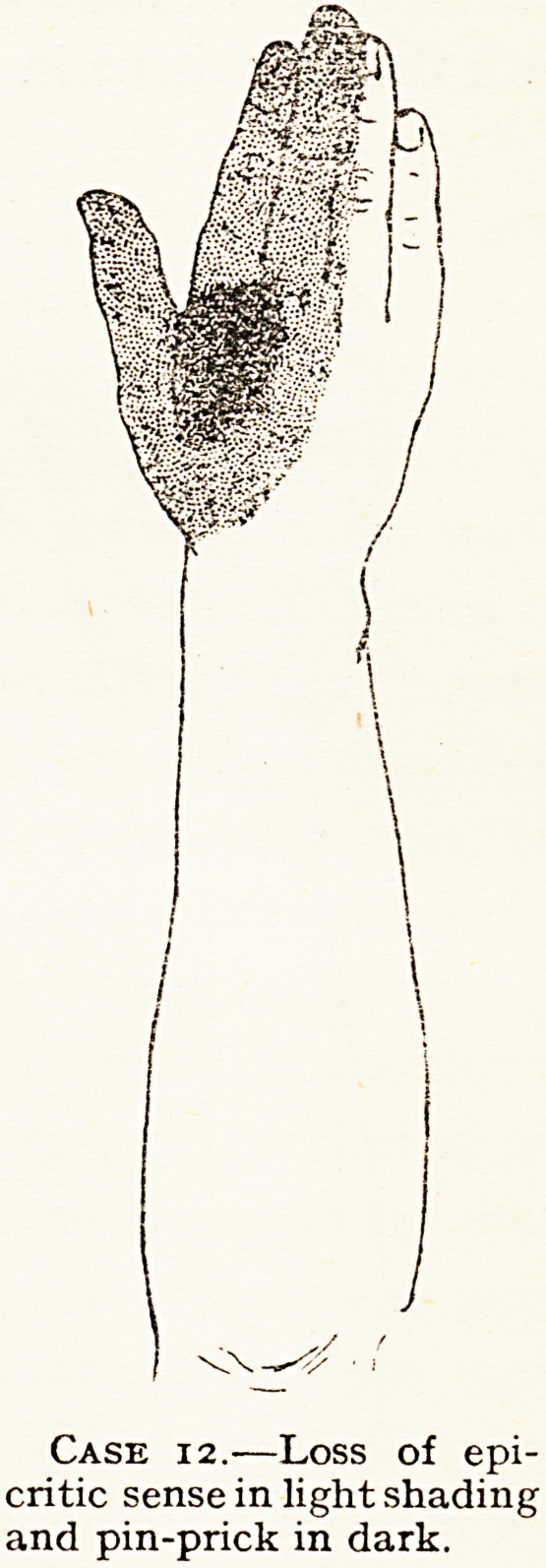


**Case 27. f8:**
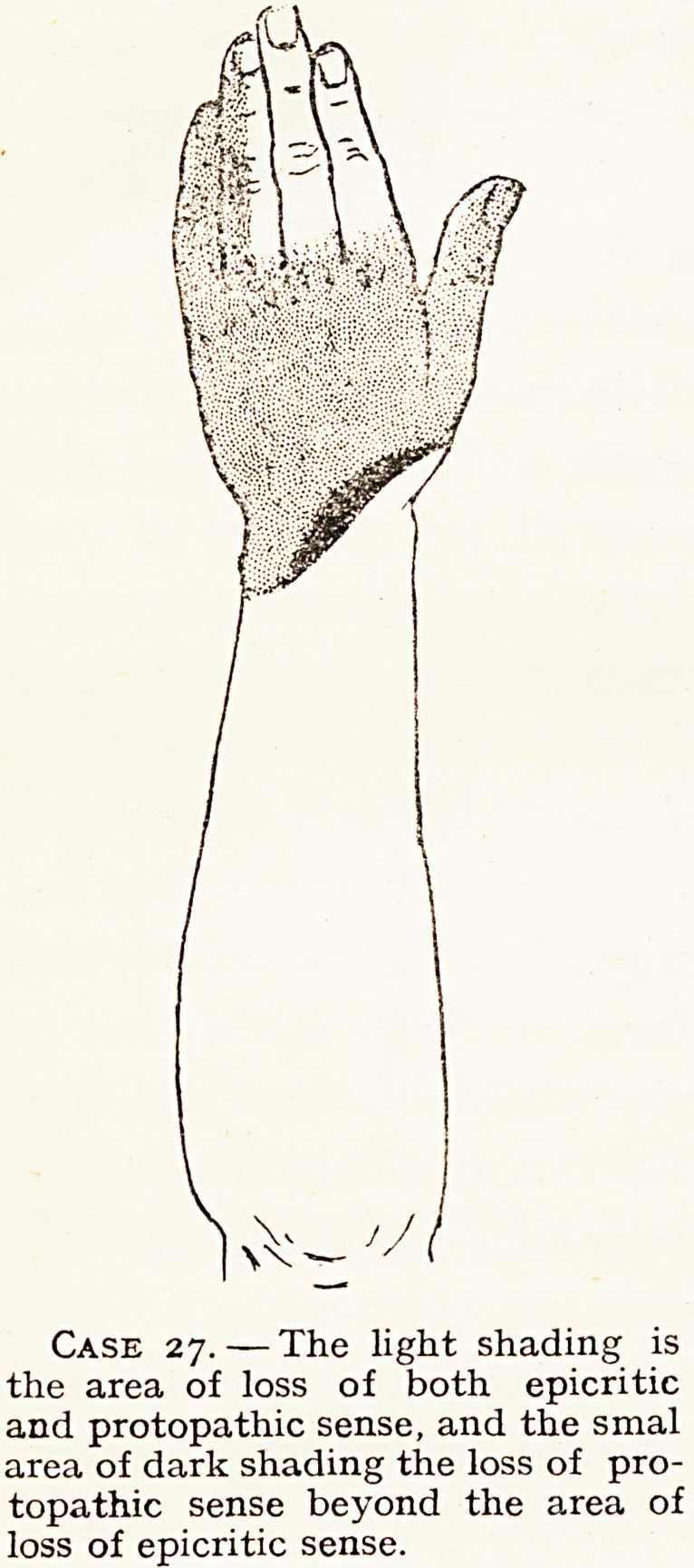


**Case 29. f9:**
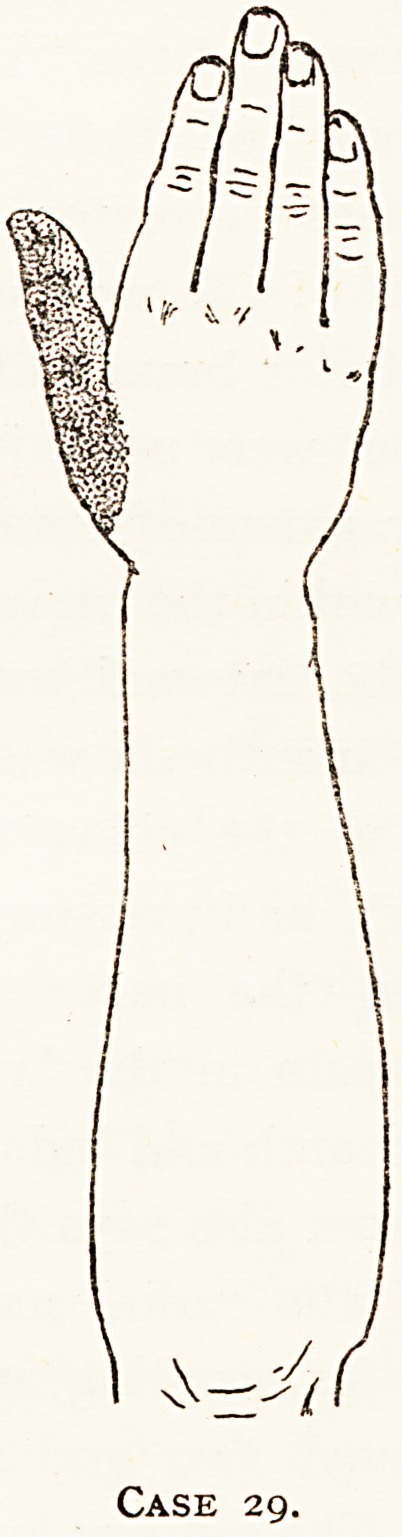


**Case 21. f10:**
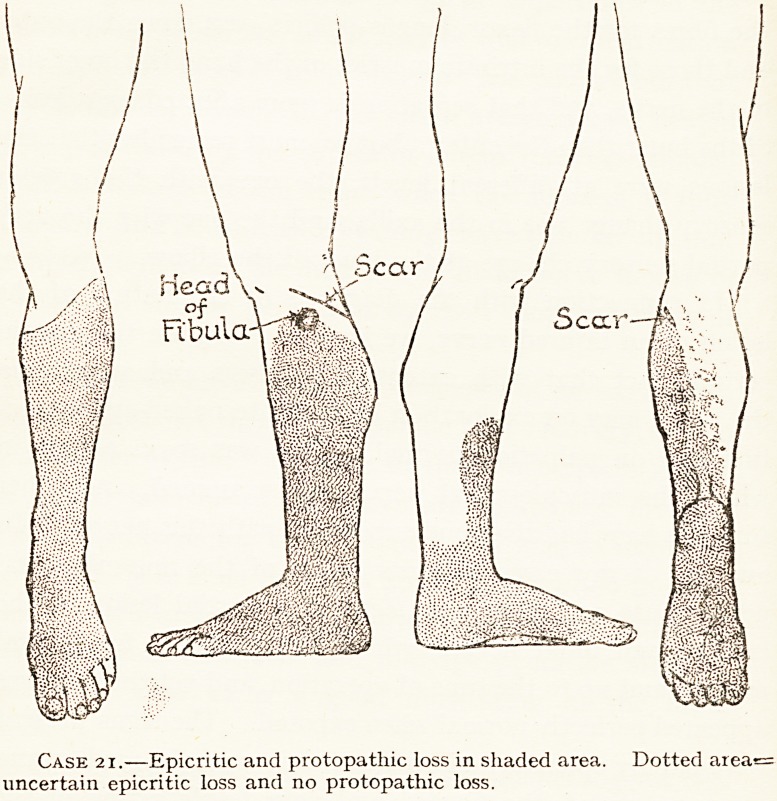


**Case 31 f11:**